# Dichotomy in the Epigenetic Mark Lysine Acetylation is Critical for the Proliferation of Prostate Cancer Cells

**DOI:** 10.3390/cancers7030854

**Published:** 2015-08-19

**Authors:** Ravi Pathak, Marc Philizaire, Shiraz Mujtaba

**Affiliations:** 1 Department of Structural and Chemical Biology, Mount Sinai School of Medicine, 1425 Madison Ave, New York, NY 10029, USA; E-Mail: ravipathak@aol.in; 2 Medgar Evers College, City University of New York, 1638 Bedford Ave, 403D, Brooklyn, NY 11225, USA; E-Mail: marc.philizaire@student.mec.cuny.edu

**Keywords:** prostate cancer, androgen, androgen receptor, lysine acetylation, *de*acetylation and molecular interactions

## Abstract

The dynamics of lysine acetylation serve as a major epigenetic mark, which regulates cellular response to inflammation, DNA damage and hormonal changes. Microarray assays reveal changes in gene expression, but cannot predict regulation of a protein function by epigenetic modifications. The present study employs computational tools to inclusively analyze microarray data to understand the potential role of acetylation during development of androgen-independent PCa. The data revealed that the androgen receptor interacts with 333 proteins, out of which at least 92 proteins were acetylated. Notably, the number of cellular proteins undergoing acetylation in the androgen-dependent PCa was more as compared to the androgen-independent PCa. Specifically, the 32 lysine-acetylated proteins in the cellular models of androgen-dependent PCa were mainly involved in regulating stability as well as pre- and post-processing of mRNA. Collectively, the data demonstrate that protein lysine acetylation plays a crucial role during the transition of androgen-dependent to -independent PCa, which importantly, could also serve as a functional axis to unravel new therapeutic targets.

## 1. Introduction

Prostate cancer (PCa), which has lately emerged as one of the most frequently detected forms of cancer, remains the second leading cause of death amongst men [1]. Though surgical and radiological interventions are standard clinical strategies, the lack of long-term treatment options necessitates the identification of new therapeutic targets to abrogate recurrence as well as metastasis of prostate tumor cells [2]. Growth of the normal prostate gland as well as PCa is explicitly dependent on the functional axis of androgen and androgen receptor (AR) [3]. Logically, most hormonal therapies target suppression of AR functions, however, despite initial success, the tumor cells acquire resistance and continue to grow again through the restoration of complex AR signaling [4]. Although a few clinical strategies could temporarily thwart recurrent and advanced stage PCa, institution of a universally effective method to completely block PCa growth is still needed. Analysis of microarray data certainly identifies changes in a target gene expression, which is indeed crucial, but it does not factor the role of epigenetic modifications, which also play a pivotal role during cell fate decisions [5]. Most recent studies have already established the overarching significance of epigenetic modifications, which confer enormous plasticity to cellular proteome and gene regulatory machinery to selectively modulate physiological processes during the development of normal prostate and PCa [6,7]. Collectively, microarray data indeed quantitatively present the level of cellular transcripts, but it is vital to understand the mechanistic underpinnings of the impact of epigenetic modifications on gene expression and expanded functions of target proteins. 

Since AR is a ligand-dependent DNA-binding transcription factor that governs downstream genes to facilitate growth of normal as well as tumor prostate tissue, the role of transcriptional coactivators become critical in modulating AR functions. Transcriptional coactivators act through multiple mechanisms that include physical interactions with general transcription factors, RNA polymerase II, epigenetic modifications of chromatin and ATP-dependent chromatin remodeling [8]. Some of the key transcriptional co-activators are the p160 family (SRC-1, GRIP1/TIF2, RAC3/pCIP/ACTR/AIB1/TRAM1), PCAF, CBP and p300 possessing intrinsic histone acetyltransferase (HAT) activity, which can be directed towards histone as well as other proteins [9,10]. AR undergoes acetylation at a conserved lysine motif ^629^RKLKK^633^ by coactivator p300 and *de*acetylation by NAD-dependent histone *de*acetylases Sirtuin 1 (Sirt1) [11,12]. A recent study demonstrates that transcriptional coactivator MYST1 (MOZ, YBF2 and SAS2, and TIP60 1) concomitantly regulates the functions of AR and Nuclear Factor-Kappa B (NF-κB) to promote aggressive proliferation and to block apoptosis of PCa cells. Interestingly, mutually exclusive interactions between *auto*acetylated MYST1 and Sirt1 play a critical role in progression of PCa cells from androgen-dependent to -independent PCa. Clearly, *auto*acetylation and *de*acetylation of MYST1 by Sirt1 have the ability to synergize AR and NF-κB pathways that can resist the impact of anti-PCa treatment [13]. 

The biochemical landscape of lysine acetylation has expanded from chromatin to a multitude of cytosolic and mitochondrial proteins [14,15]. Since the first report, which demonstrated that acetylation of the tumor suppressor protein p53 by ubiquitous HAT, CBP, is crucial for cellular response, the number of non-histone substrates of HATs, including AR has tremendously increased [16,17]. These new substrates are not limited to metabolic enzymes, cytoskeletal proteins, molecular chaperones, ribosomal proteins and nuclear import factors [14,15]. Previously, analysis by DAVID 6.7 showed that lysine acetylated proteins form functional clusters into cell signaling, stress response, proteolysis, apoptosis, metabolism, and neuronal development [14,15]. Accumulating studies continue to demonstrate that lysine acetylation plays a widespread role in the pathogenesis of many diseases, including diabetes, obesity, inflammation and cancers [13,18,19,20]. AR undergoes acetylation within the hinge region, particularly, ^629^RKLKK^633^, which is required for nuclear localization, and transcriptional activation of AR leading to cellular proliferation [17,21,22,23,24,25,26,27,28]. These data underscore that blocking acetylation could abrogate the functions of AR involved in activating the downstream molecular events, which support growth of PCa. Given the cellular wide role of lysine acetylation, the main focus of the present study is to unravel the dynamics of acetylation and *de*acetylation during the transition of androgen-dependent to -independent PCa. It is highly likely that identifying proteins undergoing acetylation in advanced PCa cells will unravel potential therapeutic targets, which subsequently, based on their abilities to block PCa growth could serve to develop future therapeutic regimes. Towards this goal, computational tools provide a unique capability to analyze high throughput gene expression data particularly for identifying protein targets susceptible for undergoing epigenetic modifications. 

## 2. Results 

### 2.1. Structural Basis for the Transcriptional Functions of AR

**Figure 1:**
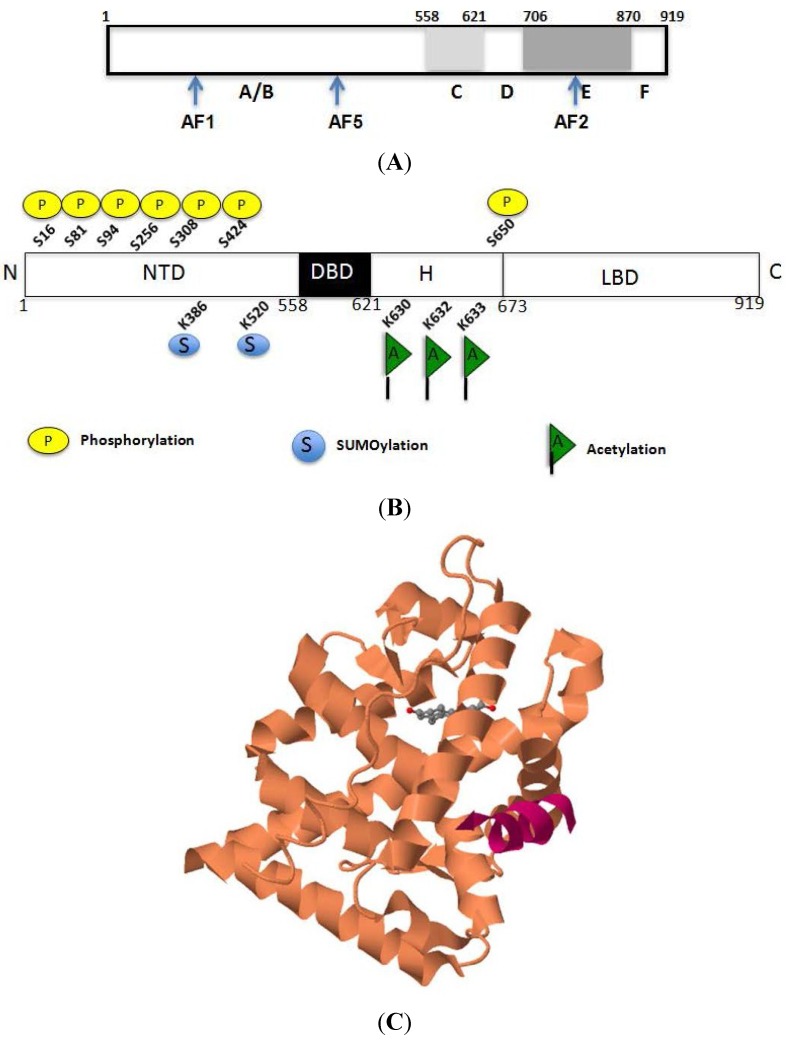
A representative Structure of AR Protein. (**A**) AR structure with N-terminal regulatory domain (A/B), activation domains (AF1 and AF5), DNA binding domain (C), Hinge region (D), ligand binding domain (E) and C terminal tail (F); (**B**) Spectrum of epigenetic modifications on AR that includes phosphorylation, acetylation and sumoylation; (**C**) Crystal structure of the human androgen receptor ligand-binding domain bound with an androgen receptor *NH2*-terminal peptide AR20-30 and R1881.

Androgens not only play a central role in male sexual differentiation, but also during the development and maintenance of the secondary male characteristics [29]. The biochemical actions of testosterone and 5α-dihydrotestosterone (DHT) are mediated by their inherent nature to interact with AR (Nuclear Receptor subfamily 3, group C, gene 4; NR3C4) [30]. Structural and functional data reveal that most nuclear hormone receptors, including AR, are comprised of a *N*-terminal regulatory domain (NTD), a DNA-binding domain (DBD), a small hinge region (H) and a ligand-binding domain (LBD) (Figure 1A,B) [31,32]. The NTD induces transcription through its two independent activation function (AF) domains, AF-1 and AF-5, which have been identified within its 558 amino acids (Figure 1A,B). While AF-1 regulates the *trans*activity of the full-length AR, AF-5 is involved in the *trans*activity of a constitutively active AR, which lacks its LBD. Recent structural observations suggest that NTD undergoes a conformation change upon binding to proteins or to DNA, raising the possibility that the NTD serves as a scaffold for the recruitment of co-regulators and assembly of the transcriptional machinery; thereby, serving as a primary mediator of the cell- and gene- specific effects of androgens [33]. The cysteine-rich DBD is comprised of two zinc-finger motifs and a short C-terminal extension that is linked to the hinge region [34,35]. One of the zinc-fingers mediates DNA recognition through its interaction with AR response elements [36], while the second zinc finger stabilizes DNA bound receptor complex and mediate dimerization between AR monomers [35,37,38]. The hinge region situated between the DBD and LBD contains a bipartite nuclear localization signal and important sites for phosphorylation, acetylation and degradation [17,39,40,41,42]. The LBD mediates high affinity binding of the AR to androgenic ligands and is formed by the ordered arrangement of 12 conserved alpha helices (Figure 1C) [43,44,45,46]. Mutation or deletion of the AF-2 domain within LBD dramatically reduces the transcriptional activation in response to a ligand [47,48]. Studies have also revealed that the NTD could compete for AF-2 surface with certain coregulators, thus, providing a mechanism for divergent determinants of AR transcriptional activities [49]. Together, multi domain structure aids in the transcription functions of AR, which depend upon binding to ligand as well as target gene promoter and coactivators to ultimately facilitate the growth of prostate gland. 

### 2.2. Acetylation-Mediated Molecular Interactions of AR

In most target tissues including the prostate, testosterone is converted into DHT [50,51], which immediately enhances its potency to bind to AR with a slower rate of dissociation. In the absence of ligand, AR is cytosolic where it exits as a complex with the Heat Shock Protein (HSP) family, such as Hsp90, Hsp70, and Hsp56 [52]. Upon binding to androgens, the AR undergoes a conformational change that releases HSP, thereby facilitating its own nuclear translocation concomitant with increased phosphorylation and acetylation, and *homo*dimerization, which leads to activation of downstream target genes. Specifically, the AR dimer binds to androgen-response elements consisting of consensus palindromic element composed of two core 5′-AGAACA-3′ motifs separated by a 3-bp space (5′-GAACANNNTGTTCT-3′) located in the regulatory regions of target genes [53,54]. Subsequently, AR actively recruits essential coactivators and assembles the transcriptional machinery, which is required to regulate the expression of androgen-regulated genes [55,56]. Other studies have suggested that dimerization of the AR occurs only after nuclear translocation, which may require prior binding to DNA [57]. Transcriptional activity of AR is directed mainly through an activation function domain, within NTD.

**Figure 2:**
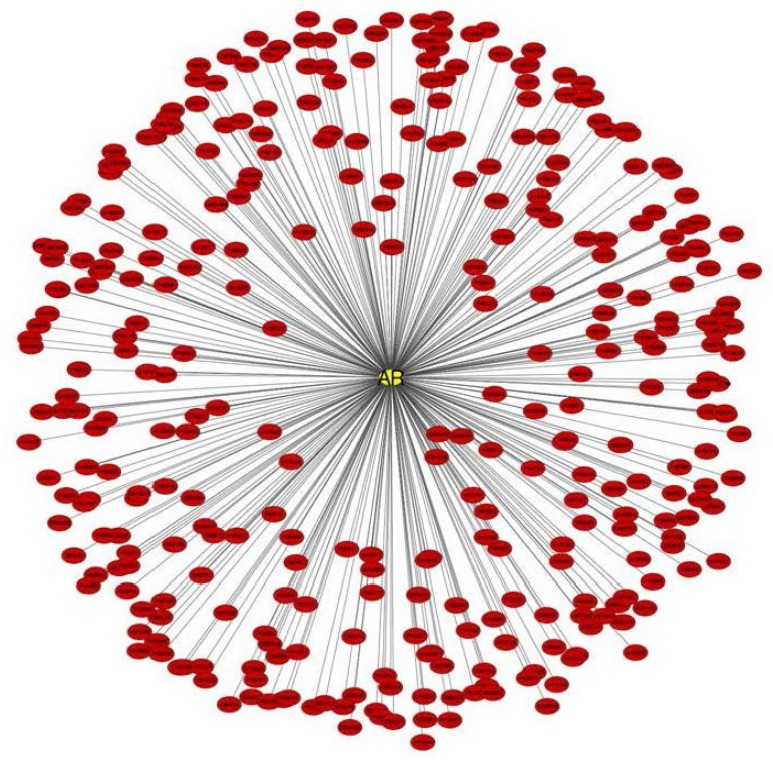
Network of AR Interacting Proteins. The AR primary interactome was compiled using manual data curation and various software including Database of Interacting Proteins (DIP), Interologous Interaction (I2D) Database, InnateDB, IntAct, MatrixDB, The Molecular INTeraction Database (MINT), Molcon, The Microbial Protein Interaction Database (MPID), Uniprot, Simons Foundation Autism Research Initiative (SFARI). AR interactome was visualized as a network using Cytoscape.

**Figure 3:**
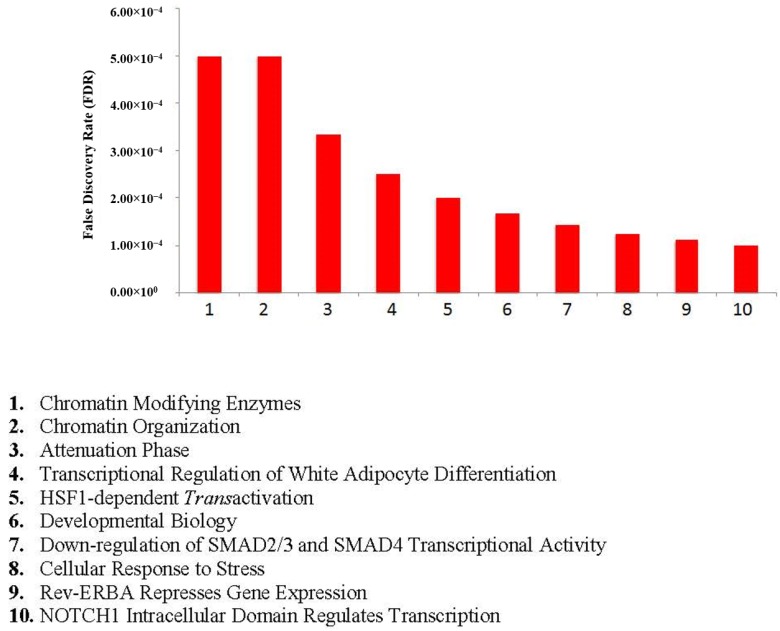
Lysine-acetylated AR-Interacting Proteins Participate in Chromatin Organization. Ninety two AR-interacting proteins were identified as being lysine acetylated. These 92 proteins were analyzed using the Reactome F1 plugin in Cytoscape. The top ten statistically relevant pathways depict the diversity of biological pathways regulated by lysine acetylated AR-interacting proteins.

A search for AR interacting partners across multiple protein interaction databases reveals upto 333 interactions, which were confirmed experimentally (Figure 2 and Supplementary Table 1). These AR-interacting proteins participate in multiple biological pathways (including prostate cancer), and are indicative of the critical role that AR plays in regulating diverse biological pathways (Supplementary Table 2). Notably, 92 AR interacting proteins are also lysine acetylated and play an important role in chromatin remodeling (Figure 3 and Supplementary Table 3), and are involved in various biological processes, as well as and pathways that facilitates prostate cancer development (Supplementary Table 4), suggesting a critical role for post translational modifications in AR-mediated processes. Collectively, these data highlight the complexity during transcriptional activation of AR due to alteration in the microenvironment of prostate cells that could trigger normal as well as tumor growth.

### 2.3. Regulation of AR Transcriptional Functions by Acetylation and Deacetylation

**Figure 4:**
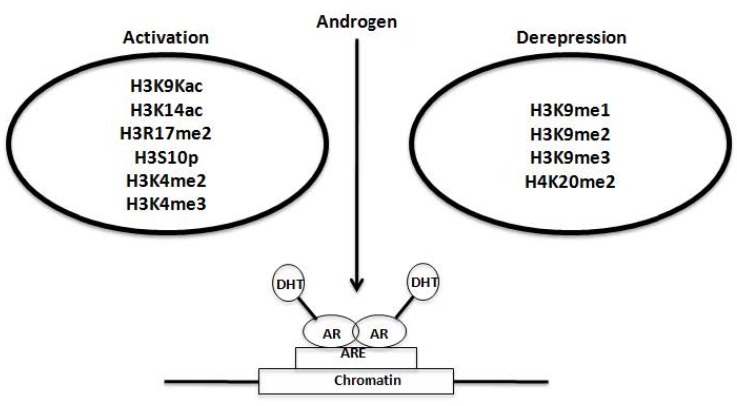
Chromatin Environment of AR Target Gene Promoter. Activating modifications include acetylation of lysine 9 and lysine 14 (H3K9ac and H3K14ac) at histone 3, *dimethylation of arginine 17(H3R17me2)*, *phosphorylation of serine 10 (H3S10p)*, *and dimethylation as well as trimethylation of lysine 4* (*H4K4me3* and me4). Apart from this *demeth*ylation of *mono*-, *di*-, and trimethyl marks at lysine 9 of histone 3 (H3K9me1, me2 and me3) and decrease in the dimethylation status of lysine 20 at histone 4 (H4K20me2) bring about removal of repressive marks.

Epigenetic modifications including acetylation, phosphorylation, methylation and sumoylation modulate AR *trans*activation function, particularly, on the target genes that control cell cycle regulatory genes (Figure 4) [27,28]. Acetylation of the AR has been shown to regulate several key functions of the AR (DNA synthesis, *trans*activation, and cellular growth), but does not affect AR-mediated *trans*repression or sumoylation [17,25,26,27,28,58]. Recruitment of histone *de*acetylase (HDAC)/NCoR/Smad complexes to a subset of cell-cycle regulatory genes, including Cyclin D1, suggests the critical role of AR acetylation [59]. Acetylation of AR is functionally associated with its phosphorylation as dephosphorylation inhibits AR activity in PCa cell lines upon the activation of the cAMP pathway, which leads to a rapid dephosphorylation of the AR likely through activation of PKA-inducible phosphatases [17,25,26,27,28,60,61]. Mutation of the lysine residues involved in acetylation reduces ligand-induced phosphorylation, while the point mutation of phosphorylation sites in the AR reduces HDAC-mediated regulation of the AR [17,25,26,27,28,60,61].

Sirt1 was recently shown to bind directly to the AR and Sirt1 expression inhibited AR activity [17,25,26,27,28,60,61]. This was further confirmed by a point mutation of the core histidine residue of Sirt1, which abrogated its *de*acetylase activity and at the same time reversed the repression of AR signaling [59,62]. Interestingly, growth suppression of PCa cellular growth by Sirt1 was observed in cells expressing the AR, but not in cells that failed to express the AR. Given the fact that Sirt1 function is regulated by DHT, it is tempting to suggest that Sirt1 is a key regulator of AR function during the development androgen-independent PCa.

### 2.4. Synergy of AR with Key Molecules to Promote Metastasis and Growth of PCa

Published studies have shown that AR is differentially expressed in most of primary PCa [63,64,65,66]. Notably, amplification of AR gene has been shown to enhance the sensitivity of PCa to the reduced levels of androgens post-ablation therapy [52], although it is unclear whether amplification of the AR gene in hormone refractory tumors results in an increase in AR protein levels. Furthermore, coregulators are the major determinant controlling transcriptional activities by promoting (coactivators) or inhibiting (corepressors) AR functions [67]. For instance, Cdc25 family of dual-specificity phosphatases activates cyclin-dependent kinases to enable cell cycle progression. The members of this family are differentially expressed in PCa, which are known to interact with AR in a ligand-dependent manner. Recently Chmelar *et al.* showed that over expression of Cdc25B in cancer cell lines and human cancers can be correlated with histological grade of prostate tumor and frequently with more poorly differentiated tumors [33]. Mutations within AR impact AR/ligand specificity, which might also contribute to the progression of PCa and the failure of endocrine therapy by allowing AR transcriptional activation in response to antiandrogens or other endogenous hormones. Furthermore, AR also induces prostate specific antigen (PSA) expression through three androgen response element-containing enhancer elements located in the proximal 6 KB of the *PSA* promoter [68,69]. PSA remains the most sensitive biochemical marker available for monitoring the prognosis of prostatic disease, particularly PCa, as well as, for determining the patients’ response to therapeutic interventions. 

One of the recent studies demonstrated that the level of BIRC6 is elevated in castration-resistant PCa [70]. Interestingly, downregulation of BIRC6 together with inhibitors of apoptosis was associated with increased apoptosis, cell cycle arrest and suppression of NF-κB activation [70]. Besides NF-κB dependent interleukin 6 pathway not only support prostate tumor survival and prevent apoptotic event but also activate AR during emergence of androgen independent PCa [71,72,73,74,75]. In advanced PCa, it is highly possible that a functional synergy between AR and the NF-κB escalates the resistance to therapeutic regimes and promotes aggressive tumor growth [13]. Although the underlying mechanisms are less clear, gene regulatory abilities of coactivators can bridge the transcription functions of AR and NF-κB. A recent study demonstrates that activation of NF-κB promotes *de*acetylation of MYST1 by Sirt1 [13]. Furthermore, the mutually exclusive interactions of MYST1 with Sirt1 *vs.* AR regulate the acetylation of lysine 16 on histone H4. Notably, in *AR* lacking PC3 cells, as well as, in AR-depleted LNCaP cells, diminution of MYST1 activates the cleavage of PARP and Caspase 3 that leads to apoptosis [13]. In contrast, in AR-transformed PC3 cells, depletion of MYST1 induces CDKN1A, which results in the arrest of cell cycle in the G2M phase [13]. Concomitantly, the levels of phospho-retinoblastoma, E2F1, CDK4 and CDK6 are reduced. Finally, the expression of Tumor Protein D52 was unequivocally affected in PC3, AR-transformed PC3 cells and LNCaP cells [13]. Collectively, these data establishes the role of histone acetyltransferases and *de*acetyltransferases, including Sirts in the pathogenesis of androgen-independent PCa. 

### 2.5. Role of Lysine-acetylated Proteins in AR-independent Progression of PCa

Castration alone or in combination with AR antagonists is routinely used for treatment of PCa. Although, initial effects of this androgen ablation results in the inhibition of AR with concomitant reduction of PSA expression [4], PCa relapses in a form that is resistant to hormonal manipulations [9]. At this particular stage, the tumor is referred to as androgen-independent, androgen-refractory, or androgen-depletion [76]. However, most androgen-independent PCa shows high levels of AR expression and PSA continues to be expressed in them [77]. Furthermore, ribozyme, antisense, and small-interfering RNA approaches have shown that targeted inhibition of the AR decreases *PSA* expression, cell proliferation, and survival in various cell-based models of androgen-independent PCa [78,79,80,81]. Several signaling pathways have been identified as being responsible for progression of PCa to androgen depletion-independent state including IGF signaling and the interplay between androgen signaling and TGF-beta [82], FGF [83,84] and VEGF [85]. However, the underlying mechanisms remain to be completely deciphered. Recent gene expression and protein arrays from LNCaP, a PCa cell line, revealed that more than 600 genes and 100 proteins were androgen-dependent [86]. This study further delineated the signaling effects that are independent of direct activation of AR and implicated that growth factor regulation of cell cycle as being one of the central mechanisms for the switch to androgen-independent proliferation in PCa. At least 565 proteins and 668 genes are identified as being androgen-independent. 

**Figure 5:**
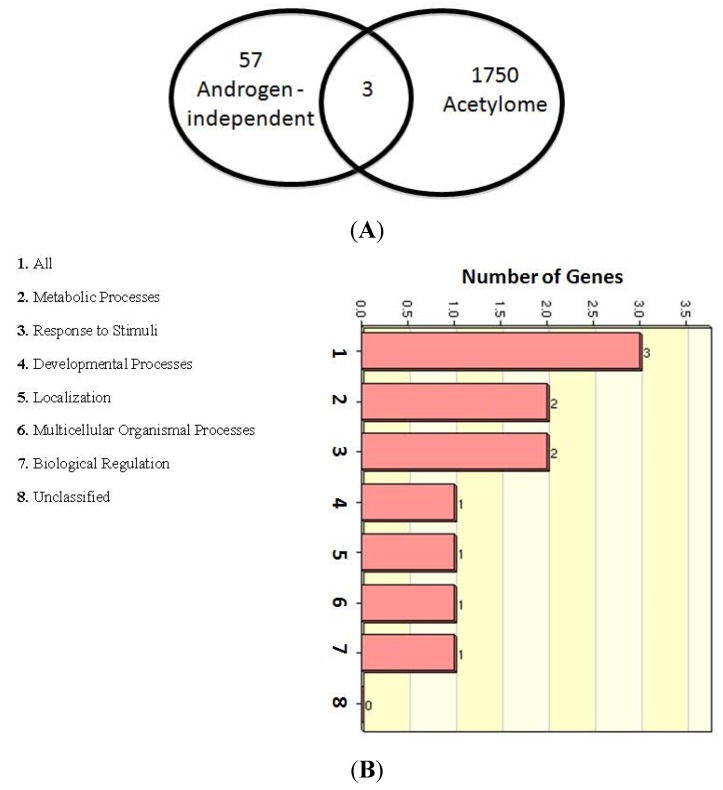
List of Top Androgen-independent Proteins Undergoing Lysine Acetylation. (**A**) Venn selection with the lysine acetylome identifies three AR-independent lysine acetylated proteins. (**B**) The proteins were analyzed using the WebGestalt software (“WEB-based GEne SeT AnaLysis Toolkit”) [87]. The Web-based Gene Set Analysis Toolkit (WebGestalt) is a suite of tools for functional enrichment analysis in various biological contexts. WebGestalt compares a user uploaded gene list with genes in pre-defined functional categories to identify those categories with enriched number of user-uploaded genes.

**Figure 6:**
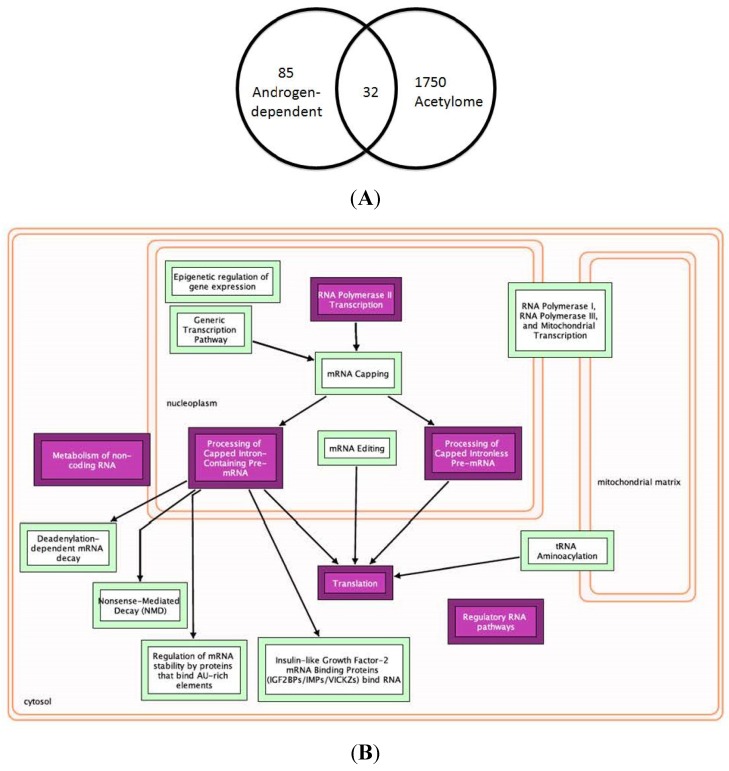
List of Top Androgen-dependent Proteins Undergoing Lysine Acetylation. (**A**) Venn selection with the lysine acetylome identifies three androgen-dependent lysine acetylated proteins. (**B**) Androgen-dependent lysine acetylated proteins regulate key pathways in mRNA processing and splicing. A list of Androgen-dependent proteins was compiled from the raw data as provided by [88]. This list was overlapped with the lysine acetylome to identify 32 androgen-dependent lysine acetylated proteins. These were further analyzed using the Reactome F1 plugin in Cytoscape to identify multiple biological pathways that are regulated by the androgen-dependent lysine acetylated proteins.

More recently, Saraon *et al.* elucidated the molecular alterations during the progression to androgen independence by comparing the proteomes of multiple androgen-independent (PC3, DU145, PPC1, LNCaP-SF, and 22Rv1) and androgen-dependent (LNCaP and VCaP) and/or normal prostate epithelial (RWPE) cell lines using mass spectrometry [88]. This study identified 57 proteins that were elevated in the androgen-independent cell lines. Our analysis from this study identified 85 proteins that were androgen-dependent (Supplementary Table 5). The results revealed that only three androgen-independent proteins were lysine acetylated (Figure 5A,B, and Supplementary Table 6), while 32 androgen-dependent proteins were lysine acetylated (Figure 6 and Supplementary Table 7).

The androgen-dependent lysine acetylated proteins were distributed between different biological processes, and pathways that include metabolism, spliceosome, and RNA processing (Supplementary Table 8). Together, these data confirm the crucial role of acetylation in advancing the metastatic abilities to PCa cells, therefore, a direct investigation to unravel the underlying epigenetic mechanisms is needed.

### 2.6. Multiple Treatment Strategies Target AR-Mediated Signaling

Chemo- and radiotherapies, androgen ablation, vaccines and AR pathway antagonists are some of the most common approaches for treatment of PCa [89,90]. Inhibition of the AR in androgen-refractory PCa cells has also been reported to shut down cell proliferation [81,91]. Studies have demonstrated that the kinase activities of PI3K and Akt are highly increased upon androgen deprivation and activation of these pathways play an essential role in the androgen-refractory progression of the PCa by stimulating cell proliferation and survival [92,93]. Bcl-2 is an androgen-regulated oncogene that is overexpressed in a variety of cancers, including androgen-refractory prostate tumors [94,95,96]. Unlike the normal human prostatic secretory epithelial cells that do not express the Bcl-2 protein [97], high levels of bcl-2 expression are detected in 30%–40% of androgen-independent cancers. Additionally, Bcl-2 expression increases with the loss of PTEN in advanced PCa [98], which leads to the assumption that androgen withdrawal and PTEN loss correlate with overexpression of Bcl-2.

Owing to the complex nature of the onset and progression of PCa, a diverse group of drugs with different targets have been tested in the recent past with varying therapeutic outcome. Therefore, a better understanding of the molecular mechanisms of androgen action combined with existing knowledge of AR signaling might offer avenues to formulate therapies against new targets leading to better therapeutic options, especially in the setting of metastatic and androgen independent PCa.

## 3. Discussion

Mann and his colleagues using high-resolution mass spectrometry discovered that at least 3600 acetylated lysine sites in 1750 cellular proteins suggesting that acetylation, like phosphorylation, is a cellular wide phenomenon [14,15]. Emerging studies have established that acetylation plays a crucial role in the pathogenesis of diabetes, obesity, inflammation, cancers and many other human diseases [13,18,19,20]. It is established that acetylation imparts transcriptional machinery the capacity to modulate cellular processes during infection, DNA damage and repair, metabolism and cell cycle progression [99,100,101,102,103]. However, the complexity in acetylation emerges due to selectivity in molecular recruitment as the acetylated-lysine moiety could mutual exclusively serves as a recruitment site(s) for bromodomain or HDACs [104]. Interestingly, these two epigenetic events profoundly impact the downstream functions, which govern cellular response and cell fate decisions. Previous studies have indicated that down-regulation of HAT containing coactivator CBP and MYST1 abrogate the growth of PCa [13,17,27,28]. Clearly, HATs and HDACs, which reversibly control the occurrence of acetyl mark could be targeted in disease models, particularly, if their knock-down leads to control of disease process. Our analysis of gene expression array data clearly showed that AR-interacting proteins as well as selected target genes/proteins in androgen-dependent as well as -independent PCa undergo acetylation. Although it is not clear which HDAC(s), drive cellular wide *de*acetylation during advancement of PCa, it certainly needs more biochemical investigations. Taken, together, it is possible that targeting HAT(s) in androgen-dependent state or targeting HDAC(s) during androgen-independent state cold have therapeutic outcomes. 

Particularly, the dynamism exhibited by epigenetic modifications, such as acetylation/*de*acetylation, which if perturbed by a small molecule has the potential to impact cell fate decisions that eventually has made them attractive targets for drug discovery program. The FDA-approved HDAC inhibitor, vorinostat, which induces the differentiation of tumor cells has been approved for treatment of T cell lymphoma [105]. Besides, several inhibitors of bromodomain showed promising results in blocking pathogenesis of cardiac ischemia and HIV infection [106,107]. Most recently, inhibitor of BRD4, JQ1, which was tested in various model systems, suppresses cMYC by binding to the first bromodomain of BRD4 [108]. Clearly, emerging studies show promising small molecules that target HDACs or bromodomain, but a selective ligand for HATs remains to be developed. Most importantly, small chemical molecule treatment of cancer cells is a gateway for not only dissecting the function of an endogenous protein with multiple functions but also could offer highly selective ligand, which could have therapeutic value. Finally, our future studies will focus to unravel the site(s) of acetylation on proteins undergoing acetylation in androgen-dependent as well as independent PCa, followed by mechanistic investigations to understand the mechanistic basis of acetylation and unravel the functional roles of these proteins during the progression of PCa pathogenesis. 

## 4. Methods of Analysis

### 4.1. Construction of AR Interactome

The AR primary interactome was compiled using manual data curation and various software including Database of Interacting Proteins (DIP), Interologous Interaction (I2D) Database, InnateDB, IntAct, MatrixDB, The Molecular INTeraction Database (MINT), Molcon, The Microbial Protein Interaction Database (MPID), Uniprot, Simons Foundation Autism Research Initiative (SFARI).

### 4.2. Network Visualizations and Reactome Analysis

Cytoscape tool was used for creating AR interactome visualizations. Additionally, the Reactome FI plugin for cytoscape was used to enrich gene sets and visualize pathways [109,110].

### 4.3. Functional Enrichment Analysis

Functional Enrichment Analysis was performed using the WebGestalt software (“WEB-based GEne SeT AnaLysis Toolkit”) [87]. The Web-based Gene Set Analysis Toolkit (WebGestalt) is a suite of tools for functional enrichment analysis in various biological contexts. WebGestalt compares a user uploaded gene list with genes in pre-defined functional categories to identify those categories with enriched number of user-uploaded genes [87].

## Supplementary Files

Supplementary File 1

## Abbreviations

PCa: Prostate Cancer
AR: Androgen receptor mainly localized to prostate cells that binds to androgen
HATs: Histone acetyltransferases that was identified first to enzymatically acetylate histone proteins also acetylate, cellular proteins including transcription factors and metabolic enzymes
HDACs: Histone *de*acetylases enzymatically removes the acetyl marks imprinted by HATs
PSA: Prostate Specific Antigen is a target gene directly regulated by the transcription factor AR. The presence of PSA beyond normal level in the blood is an indicator for altered prostate functions
